# A human infertility-associated KASH5 variant promotes mitochondrial localization

**DOI:** 10.1038/s41598-021-89439-2

**Published:** 2021-05-12

**Authors:** Sana A. Bentebbal, Bakhita R. Meqbel, Anna Salter, Victoria Allan, Brian Burke, Henning F. Horn

**Affiliations:** 1 College of Health and Life Sciences, Hamad Bin Khalifa University, Doha, Qatar; 2 Faculty of Life Sciences, University of Manchester, Manchester, M13 9PT UK; 3 Laboratory of Nuclear Dynamics and Architecture, Institute of Medical Biology, Agency for Science, Technology and Research (A*STAR), Singapore, Singapore

**Keywords:** Mitochondria, Mechanisms of disease, Protein translocation, Male factor infertility

## Abstract

KASH5 is the most recently identified member of the KASH domain family of tail anchored, outer nuclear membrane (ONM) and endoplasmic reticulum (ER) proteins. During meiosis prophase I, KASH5 and SUN1 form a complex that spans the nuclear envelope and which links the telomeres of meiotic chromosomes to cytoplasmic dynein. This connection is essential for homologous chromosome dynamics and pairing. A recent study identified a variant in human KASH5 (L535Q) that correlated with male infertility associated with azoospermia. However, no molecular mechanism was described. Here, we report that this amino acid substitution, within the KASH5 transmembrane domain (TMD) has no predicted effects on secondary structure. However, the overall hydrophobicity of the L535Q TMD, is calculated to be lower than the *wild-type* KASH5, based on the GES (Goldman–Engelman–Steitz) amino acid hydrophobicity scale. This change in hydrophobicity profoundly affects the subcellular localization of KASH5. Through a series of amino acid substitution studies, we show that the L535Q substitution perturbs KASH5 localization to the ER and ONM and instead results in mistargeting to the mitochondria membrane. We suggest that this mislocalization accounts for the infertility and azoospermia phenotype in patients.

## Introduction

The nuclear envelope (NE) is a defining feature of eukaryotic cells. By separating the genome from the cytoplasm, the NE establishes the boundary of the nucleus. The NE is composed of two highly organized lipid bilayers: the inner (INM) and outer (ONM) nuclear membranes separated by a roughly 50 nm gap or perinuclear space (PNS)^1^. The INM and ONM are connected at sites of nuclear pore complex (NPC) insertion. However, the two membranes maintain a distinct protein composition. Moreover, the ONM exhibits numerous continuities with the endoplasmic reticulum (ER) to which it is functionally related. In this way, the INM, ONM and ER represent different domains of a single continuous endo-membrane system with the PNS representing a perinuclear extension of the ER lumen. Beyond its role as a physical barrier, the NE has a pivotal role in various cellular functions including signaling pathway, transcriptional regulation, chromatin and cytoskeleton organization^2^. All these functions are mediated by an array of protein complexes present on both INM and/or ONM.

One such protein complex is the LINC complex (LInker of Nucleoskeleton and Cytoskeleton)^3^. By spanning the NE, LINC complexes form a bridge that physically connects the nucleoskeleton to the cytoskeleton. This connection plays a crucial role in mechanical force transmission to the nucleus^4,5^. LINC complexes contribute to a wide range of essential cellular functions including nuclear positioning and migration, centrosome localization, cell polarization and migration, and transduction of external and internal mechanical stimuli^4–6^. The LINC complex consists of two highly conserved transmembrane protein families: SUN (Sad1, UNC-84) domain proteins of the INM and KASH (Klarsicht, ANC-1, Syne Homology) domain proteins of the ONM. The lumenal domains of both SUN and KASH proteins associate within the PNS to form the core structure of LINC complexes^3,7–9^.

To date, five SUN domain proteins have been identified in mammals, SUN1-5. However, only two of these, SUN1 and SUN2 are widely expressed. The remaining three appear to be largely testis-specific^10^. The mammalian SUN family members all share a common topology. Their N-terminal domain resides in the nucleoplasm where it tethers nuclear structures to the INM, while their C-terminal segment, containing the highly conserved SUN domain (~ 175 residues), extends into the PNS where it engages with KASH family members^11^.

Six members of the mammalian KASH domain family of tail-anchored transmembrane proteins have been identified to date. These include Nesprin (nuclear envelope spectrin repeat domain)-1, -2, -3 and -4, KASH5 and LMRP (Lymphocyte Restricted Membrane Protein, also known as JAW1). The N-terminal domain of all KASH proteins is exposed to the cytoplasm where it may interact with one or more components of the cytoskeleton through which they transduce cytoskeletal forces to the NE. The C-terminal KASH domain is the eponymous feature of the KASH protein family. It consists of a single transmembrane region followed by a short lumenal tail (~ 30–40 residues) that associates with SUN domains in the PNS where a single SUN trimer can associate with three KASH peptides forming a SUN-KASH 3:3 assembly^7,12^. Recently, it has been suggested that two 3:3 complexes may associate together “back-to-back”, giving rise to a branched 6:6 SUN-KASH assembly. This larger complex could explain how the LINC complex provides for maximal forces transfer across the NE^13^.

LINC complex assembly occurs in an orchestrated fashion. The first step involves the translocation of SUN proteins from the ER/ONM to the INM via the NPC membrane domain. Once at the INM, SUN protein localization is likely stabilized by interactions with nuclear components, including the NE-associated nuclear lamina. Oligomerization also potentially plays a part in stabilizing the INM localization^7,14,15^. The positioning of SUN proteins at the INM is prerequisite for the recruitment of KASH domain proteins^16^. Various studies have shown that the KASH domain sequence is necessary and sufficient for the targeting of KASH proteins to the ONM^16,17^. While it is assumed that KASH insertion follows the same pathways as other tail-anchored proteins, the exact mechanism for KASH protein insertion into the ER/ONM has not been formally established^11^.

Tail-anchored proteins represent 3–5% of all transmembrane proteins^18^. The hallmark of the TA proteins is a single transmembrane domain (TMD) close to the C-terminus, which acts as a targeting signal. Because of its proximity to the C-terminus, it only emerges from the ribosome tunnel after translation has been terminated^19^. As a consequence, TA protein sorting and insertion occurs, by definition, post-translationally. In mammals, the mechanism(s) of TA protein insertion into membranes is not well understood. It is generally considered that the pathway responsible for membrane integration depends on the subcellular destination where the protein is to be inserted. For instance, protein translocation into the ER membrane requires TRC40 (TMD Recognition Complex), a mammalian ortholog of the yeast GET protein pathway^20–22^. Similarly, the delivery of TA proteins to mitochondria and peroxisomes involve TOM and PEX19/PEX3 machinery, respectively^23,24^. In addition, several studies have shown that the destination of a protein is not the only factor that determines what import machinery is involved. Furthermore, in vitro experiments indicate that some TA proteins are able to insert into membranes without assistance of any translocation machinery. This spontaneous insertion is correlated with the TMD hydrophobicity and the presence of charged residues in the protein tail^25–29^. Whether spontaneous insertion can occur in vivo is still an open question.

KASH5 (CCDC155) is the most recently identified KASH domain protein family member. A yeast two-hybrid screen of a mouse testis library using Shugosin2 as bait fortuitously detected KASH5^30^. Independently, KASH5 was also identified through a homology-based approach that showed a similarity between CCDC155 and the zebrafish LRMP homologue, Fue^31^. In mammals, KASH5 is found predominantly in testes and ovaries where it functions as an ONM adapter for cytoplasmic dynein. In the testis, KASH5 expression is restricted to primary spermatocytes where, in association with SUN1, it is essential for meiotic prophase I progression^31,32^. Homologous chromosome pairing, synapsis and recombination requires two important events: (1) the attachment of telomeres to the INM and (2) rapid, telomere-led chromosome movements. The KASH5/SUN1 LINC complex is responsible for both phenomena. SUN1 provides a tether for telomeres at the INM while at the same time anchoring KASH5 in the ONM^33^. KASH5 in turn transmits dynein-generated forces to telomeres via SUN1. Nuclear and autonomous chromosomes movements, called rapid prophase movement (RPM) occur to facilitate chromosome pairing and to separate or prevent non-homologous chromosome interaction^32,34,35^. RPM is driven by SUN1/KASH5 complexes that couple the telomeres to the microtubule system via cytoplasmic KASH5-dynein interactions^32^. Since correct homologous chromosome pairing is essential for meiosis, the prophase I progression is subject to stringent quality control mechanisms that monitor chromosome pairing and integrity^36–38^. Any defects or chromosomal abnormalities lead to a meiotic delay or to an early meiotic arrest, and consequently a failure in gametogenesis^39^. Indeed, mice deficient for KASH5 exhibit expected defects in RPM and homologous pairing^31,32^, with spermatogenesis arrested at leptotene/zygotene stage. Consequently, KASH5-null mice are sterile and display azoospermia^31^.

A recent study has identified a variant in human KASH5 correlated with infertility^40^. The KASH5 homozygous variant was found in two brothers affected by azoospermia, a severe type of male infertility characterized by a complete absence of spermatozoa production due to a spermatogenesis arrest. The identified variant consists of a substitution of a leucine with a glutamine at the position 535 (L5353Q) within the KASH5 transmembrane domain^40^. The molecular mechansism underlying this infertily have not been described.

In this study, we characterized the human KASH5 L535Q variant. We show that the amino acid substitution leads to a change in the transmembrane domain hydrophobicity. Using a number of KASH5 expression constructs we demonstrate that the moderate hydrophobic TMD of L535Q KASH5 resulted in a relocalization away from the NE, with a clear localization to the mitochondria. We suggest that this functional loss of KASH5 from the ONM is responsible for the azoospermia phenotype of these patients.

## Results

### The L535Q variant changes the transmembrane domain hydrophobicity of KASH5

**Figure 1:**
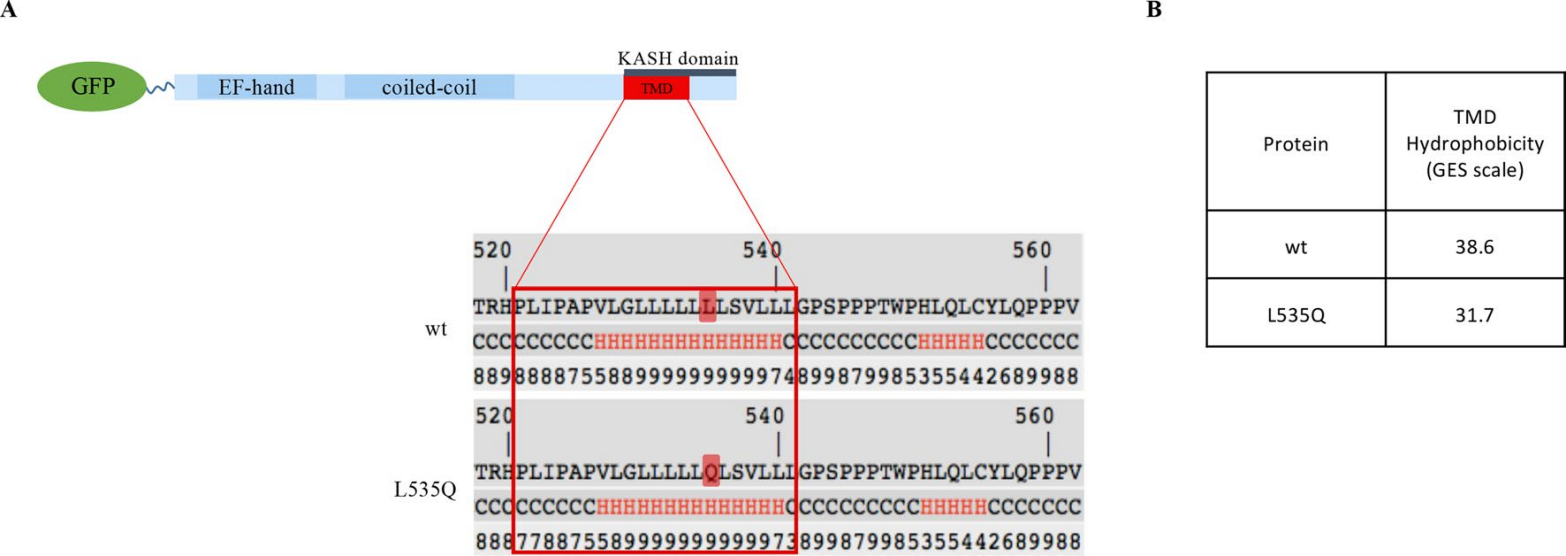
The L535Q KASH5 variant changes the TMD hydrophobicity (**A**) Schematic representation of KASH5 protein. The variant amino acid is situated in the C-terminal transmembrane domain, part of the KASH domain. The variant consists in a single amino acid substitution in the 7-leucine stretch resulting in the replacement of a leucine (L) with a glutamine (Q) at the position 535 (L535Q). According to I-TASSER (University of Michigan), wild-type and L535Q KASH5 share the same predicted helical secondary structure within the TMD (H: Helix; C: Coil). (**B**) Using the GES scale, the TMD hydrophobicity of the L535Q variant is lower than that of the wild-type KASH5 (kcal/mol).

The human *KASH5* variant that is associated with infertility is a T > A transversion at position 1604 in the cDNA^40^. This base substitution results in an amino acid replacement of a Leucine with a Glutamine, (L535Q) within the KASH5 transmembrane domain (TMD, Fig. 1A). To understand the effects of this substitution we compared the predicted secondary structure (I-TASSER, University of Michigan) of the TMDs for both the wild-type and L535Q variant KASH5 proteins. These analyses indicated that the L535Q substitution is not expected to perturb the alpha-helical structure of the KASH5 TMD (Fig. 1A). We then examined the hydrophobicity of the TMD using the GES scale (Goldman–Engelman–Steitz)^41^. At a hydrophobicity of 31.7 kcal/mol, the TMD of the L535Q KASH5 is clearly less hydrophobic than the wild-type KASH5 (38.6 kcal/mol), a reduction of 6.9 kcal/mol (Fig. 1B).

### L535Q KASH5 localizes to the mitochondria

Several studies have shown that TMD hydrophobicity and tail charge can influence TA protein targeting to specific organelles^25,42,43^. A moderate hydrophobic TMD in conjunction with a positively charged tail targets TA proteins to the outer mitochondria membrane (OMM) or to the peroxisome membrane^43,44^.

**Figure 2:**
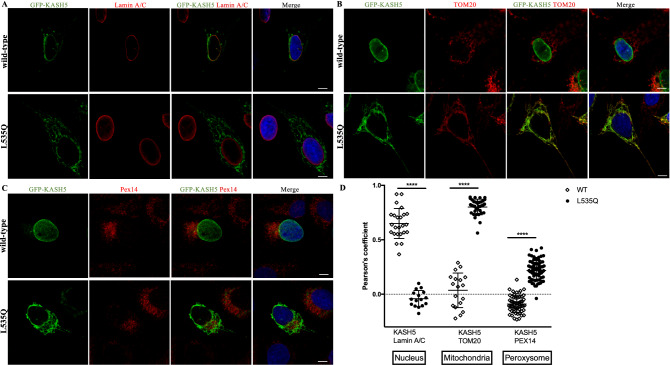
The L535Q KASH5 localizes to the mitochondria. U2OS cells grown on coverslips were transfected with an N-terminal GFP-tagged version of wild-type or L535Q KASH5. The subcellular localization was determined by immunostaining with organelle-specific protein markers. (**A**) Nuclear localization was assessed using an anti-lamin A/C antibody (red). (**B**) Mitochondria localization was assessed with an anti-TOM20 antibody (red). (**C**) Peroxisome localization was assessed with an anti-PEX14 antibody (red). (**D**) Pearson’s correlation coefficient between GFP-KASH5 and organelle markers. KASH5 vs Lamin A/C: N = 24, KASH5 vs TOM20: N = 35 and KASH5 vs Pex14: N = 72. The values indicate − 1: opposing, 0: no and 1 complete colocalization. Scale bar 10 μm. ****P < 0.0001; ***P < 0.0002. The experiment was repeated seven times.

Given the decreased hydrophobicity of the L535Q KASH5 TMD, we examined whether this change in hydrophobicity could cause a mitochondrial or peroxisomal localization. GFP-tagged versions of wild-type and L535Q KASH5 were expressed in U2OS cells and organelle localization was examined by immunofluorescence microscopy in conjunction with organelle-specific protein markers (Fig. 2). As expected, the wild-type KASH5 protein colocalized with Lamins A and/or C, components of the nuclear envelope. In contrast, the L535Q variant showed no evidence of targeting to the NE (Fig. 2A). Instead, the L535Q KASH5 showed very obvious colocalization with the mitochondrial protein, TOM20 (Fig. 2B). Evidently, L535Q KASH5 is mistargeted to mitochondria. This effect was not cell-specific since mitochondrial localization of the L535Q KASH5 was also observed in HeLa, MCF-7 and MRC-5 cells (Supplementary Fig. S1). Significantly, no colocalization was found between the L535Q KASH5 and the peroxisomal protein, Pex14 (Fig. 2C). To confirm our confocal images data, we performed a pixel-wise Pearson’s colocalization test to quantify the overlap between the GFP-KASH5 proteins and the organelle markers: Lamin A/C, TOM20 or Pex14 (Fig. 2D). A Pearson’s correlation coefficient value of 1 indicates complete colocalization, 0 no colocalization and − 1 opposing localizations. This analysis confirmed that the GFP-L535Q KASH5 did not colocalize with Lamin A/C, with a Pearson’s correlation coefficient of − 0.05. The Pearson’s correlation coefficient for the GFP-L535Q KASH5 and Pex 14 was 0.2, which is significantly higher than the correlation coefficient for wild-type KASH5 and Pex 14. We suggest that this is an artefact due to cross-over in three-dimensional space between mitochondria and peroxisomes, which appears as colocalization in a 2D image analysis. In our visual inspection of 70 + images we were unable to positively identify peroxisomal localization of L535Q KASH5. By contrast, GFP-L535Q KASH5 and TOM20 showed clear colocalization, with a Pearson correlation coefficient of 0.81, confirming a mitochondrial targeting of the L535Q KASH5. Further analyses on isolated mitochondria from U2OS cells transfected with wild-type or L535Q KASH5 showed the presence of the L535Q KASH5 protein at the mitochondria fraction (Supplementary Fig. S1D). Clearly, the amino acid change within the L535Q TMD results in the redirection of KASH5 from the ER/ONM to mitochondria.

### TMD hydrophobicity determines KASH5 localization

**Figure 3:**
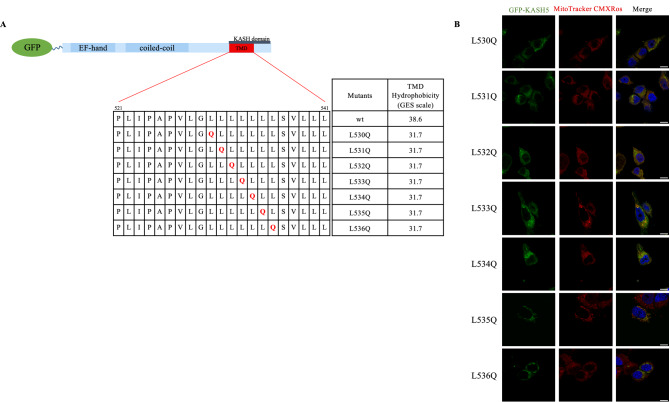
The position of the glutamine in the 7-leucine stretch does not disrupts the protein localization. (**A**) A schematic of KASH5 TMD indicating the sequential replacement of a leucine with a glutamine in the 7-leucine stretch. Computed according to the GES scale, all the variants have the same hydrophobicity of the TMD. (**B**) MCF-7 cells transfected with a N-terminal GFP-tagged version of KASH5 variants and treated with MitoTracker Red CMXROS to stain the mitochondria. All substitutions within the 7-leucine stretch lead to the mislocalization at the mitochondria. Scale bar 10 μm. The experiment was repeated two times.

The KASH5 TMD contains a stretch of seven leucine residues. The L535Q substitution is located at the sixth position of this string of leucines. To differentiate between sequence-specific requirements versus change in hydrophobicity, we sequentially altered the 7-leucines to glutamine, from position 530 to 536, as illustrated in Fig. 3A. On the GES scale, all variants have the same change in TMD hydrophobicity compared to the wild-type ($\Delta$6.9 kcal/mol). GFP-tagged variants were transfected into MCF-7 cells and analyzed by immunofluorescent microscopy. All L-to-Q TMD variants of KASH5 showed a similar subcellular localization to the L535Q KASH5 (Fig. 3B), indicating that a change in hydrophobicity is dominant over specific TMD sequence requirements in promoting L535Q localization away from the NE.

**Figure 4:**
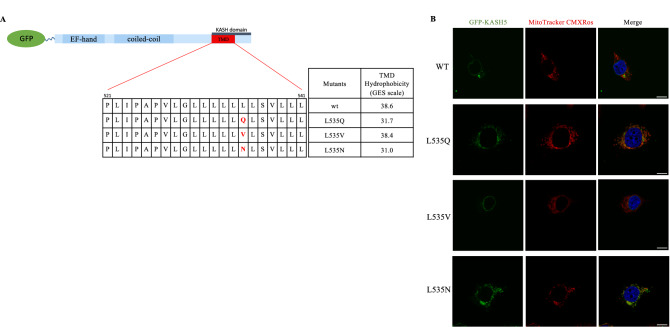
Hydrophobicity of the transmembrane domain alters KASH5 protein localization. (**A**) Schematic representation of KASH5 TMD indicating the different amino acid substitution at the position 535 (V, valine; Q, glutamine; N, asparagine). Using the GES scale to calculate the hydrophobicity, the L535V and L535N substitutions mimic the hydrophobicity of the TMD of the wild-type and L535Q KASH5, respectively. (**B**) MCF-7 cells transfected with a GFP-tagged version of wild-type or variant KASH5 and treated with MitoTracker Red CMXROS to stain the mitochondria. Both wild-type and L535V KASH5 localize at the nuclear envelope while both L535Q and L535N localize at the mitochondria. This experiment was repeated four times.

To further confirm that the change in hydrophobicity determines the mitochondrial mislocalization of the L535Q variant, we substituted the L535 with valine or asparagine, L535V or L535N, respectively. The valine substitution confers minimal change in TMD hydrophobicity compared to the wild-type TMD $(\Delta$0.2 kcal/mol, Fig. 4A). By contrast, the asparagine substitution confers a TMD hydrophobicity similar to the L535Q variant ($\Delta$0.7 kcal/mol compared to L535Q, $\Delta$7.6 kcal/mol compared to wild-type, Fig. 4A). MCF-7 cells were transfected with GFP-tagged variants or wild-type KASH5, while mitochondria were visualized with MitoTracker Red CMXROS. As revealed in the Fig. 4B, the L535V subsitution behaved in a way that was similar to the wild-type KASH5, with no evidence of targeting to mitochondria. In contrast, the L535N substitution displayed a mitochondrial localization that was indistinguishable from the L535Q KASH5 variant.

Taken together, these results suggest that the KASH5 mislocalization is not sequence dependent, but it is caused by reduced hydrophobicity of the TMD.

### L535Q KASH5 does not impair the mitochondrial membrane potential

**Figure 5:**
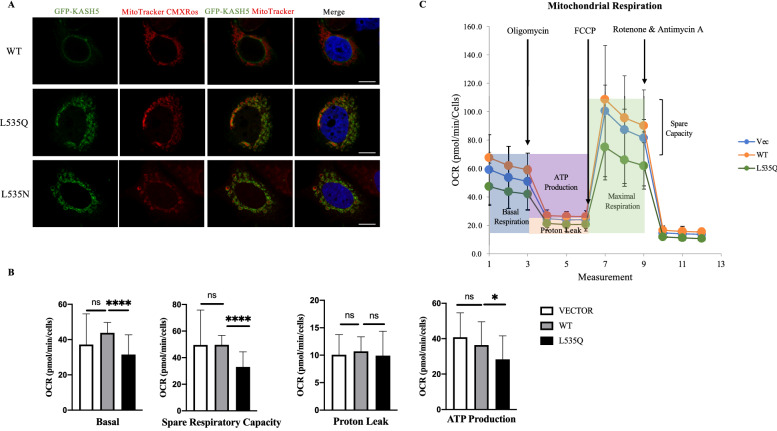
L535Q KASH5 reduces mitochondrial function, but does not impair membrane potential. (**A**) Colocalization between Mitotracker Red CMXROS and L535Q or L535N KASH5. Scale bar 10 μm. (**B**) Mitochondrial function assessed by measuring the oxygen consumption rate (OCR) in mitochondria from U2OS cells transfected with an empty vector, wild-type or L535Q KASH5. Black arrows indicate the time of addition of each mitochondrial functional modifier: Oligomycin, FCCP, Rotenone & Antimycin A. Data are expressed as mean ± standard error. (**C**) Basal respiration, Spare respiration capacity, proton leak and adenosine triphosphate (ATP) production and spare respiratory capacity of the cells. Error bars represent standard error. P < 0.05. The results are the average of two biological replicates and 30 technical replicates for each sample.

The uptake of the MitoTracker Red CMXROS dye used for the colocalization assay is dependent upon mitochondrial membrane potential (ΔΨm). As shown in Fig. 5A, the co-staining of this dye with L535Q and L535N KASH5 proteins would imply that aberrant targeting of these proteins does not significantly compromise the ability of mitochondria to maintain their membrane potential.

To further evaluate the effect of L535Q KASH5 on mitochondrial function, oxygen consumption rate (OCR) was evaluate using the Seahorse XF instrument. As shown in Fig. 5B,C, U2OS cells transfected with L535Q KASH5 exhibited a slight change in basal respiration and a small but statistically significative decrease in spare respiratory capacity and ATP production. However, the proton leak, which is correlated to the mitochondrial damage, was not altered. This data is consistent with the confocal analysis where the L535Q variant colocalizes at mitochondria that stain positively with the membrane potential-dependent stain, MitoTracker Red CMXROS.

To further confirm that the membrane potential is maintained when the L535Q KASH5 variant is present at the mitochondria membrane, we performed staining with the membrane-potential-sensitive dye TMRE (Supplementary Fig. S2). U2OS cells were transfected with GFP-tagged L535Q and stained with TMRE. A control was treated with FCCP, an uncoupler of mitochondrial oxidative phosphorylation that results in loss of mitochondrial membrane potential, and loss of TMRE staining. The results showed that while FCCP treatment abolished TMRE staining, L535Q mitochondrial localization did not.

Taken together, these data show that while L535Q KASH5 does appear to have some effects on mitochondrial function, it is not severe enough to disrupt mitochondrial membrane potential.

### Extending the tail Domain of KASH5 does not prevent mitochondrial localization

The nature of the interaction of L535Q KASH5 with the mitochondria is not known. While the TMD mutagenesis results imply an insertion into the MOM, we sought to confirm this more directly. The KASH domain consists of the TMD and a C-terminal portion that, at the NE, extends into the perinuclear space where it interacts within the binding pocket of SUN proteins to generate a functional LINC complex. The PPPX motif located at the very C-terminus of the KASH domain is essential for binding to SUN^6,7^, and extension of this sequence by a single alanine abolishes not only the binding to SUN proteins but also the localization of KASH protein to the ONM^7^.

**Figure 6:**
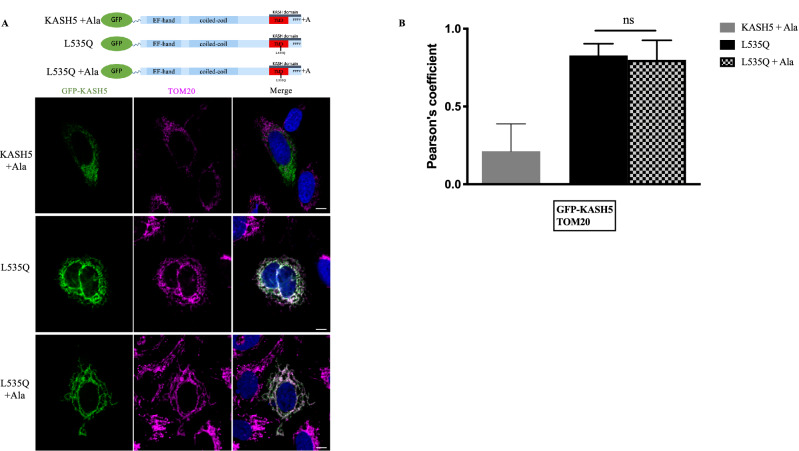
Addition of alanine to the KASH domain does not disrupt L535Q mitochondrial targeting. (**A**) U2OS cells grown on coverslips were transfected with an N-terminal GFP-tagged version of wild-type +Ala, L535Q or L535Q + Ala KASH5. Mitochondria localization was assessed with an anti-TOM20 antibody (pink). (**B**) Pearson’s correlation coefficient between GFP-KASH5 and TOM20 N = 58. The experiment was repeated four times.

We investigated whether the addition of a single alanine to the PPPX motif of the L535Q KASH5 would disrupt the mitochondria localization. A GFP-tagged L535Q KASH5 containing an extra alanine at the very C-terminus tail (L535Q + A) was transfected into U2OS cells and visualized together with anti-TOM20 staining to determine the subcellular localization. We found that the L535Q + A KASH5 localizes to the mitochondria in a fashion similar to that of the variant L535Q KASH5 (Fig. 6A). Pearson’s correlation between the GFP-KASH5 and TOM20 showed no significant difference between L535Q and L535Q + A KASH5 (Fig. 6B). In contrast, a similar extension of the C-terminus of wild-type KASH5 (KASH5 + Ala) resulted in failure of the protein to localize to the NE (Fig. 6A). A Pearson’s correlation analysis shows that KASH5 + Ala also does not localize to the mitochondria (Fig. 6B).

### L535Q KASH5 does not insert spontaneously into the lipid bilayers

**Figure 7:**
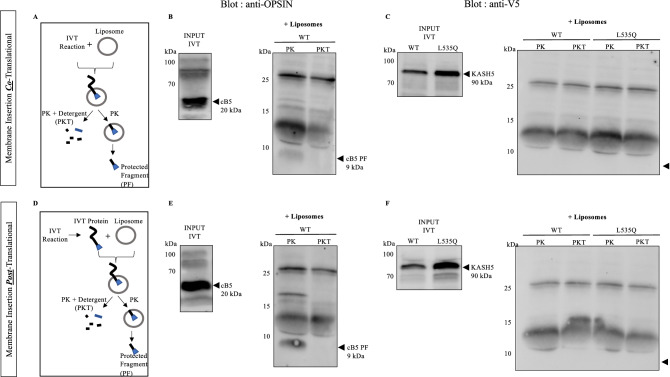
L535Q KASH5 does not insert spontaneously into protein-free liposomes. A protein-free liposome system was used to check for the spontaneous membrane insertion of L535Q KASH5. Liposomes were added either during the in vitro transcription/translation reaction [co-translational (**A**)] or after the proteins were translated [post-translational (**D**)]. Both samples were then subjected to proteinase K digestion in the presence or absence of TritonX100 detergent (PKT and PK, respectively). Co- or post-translational translocation assays were dissolved in loading buffer and subjected to immunoblot analysis with an anti-opsin or an anti-V5 antibody to detect the protected fragment (PF). For all samples, protein expression levels were determined by running an aliquot of the in vitro transcription/translation reaction (Input IVT). An opsin-tagged Cytochrome B5 was used as a positive control for spontaneous insertion into liposomes, which showed insertion in both the co-translational (**B**) and post-translational setup (**E**) as evidenced by the ~ 9 kDa protected fragment (cB5 PF). For wild-type and L535Q KASH5 proteins, the expected size of the protected fragment is also ~ 9 kDa. Neither wild-type nor L535Q KASH5 showed any spontaneous insertion activity, either co-translationally (**C**), or post-translationally (**F**). The experiment was repeated five times.

Recent in vitro studies have demonstrated that TA proteins with a moderate TMD hydrophobicity are capable of unassisted, spontaneous insertion into lipid bilayers^25,27,28,43^. Since the L535Q substitution reduces the KASH5 TMD to a hydrophobicity value that would be consistent with such proposed behaviour, we sought to determine whether L535Q KASH5 can spontaneously insert into membranes. To prevent the cellular machinery from contributing to the insertion, we used a protein-free liposomes system (Fig. 7). The KASH5 constructs were tagged at the C-terminus with V5 epitope tag. This allowed us to cleave any extra-liposome moiety and retain enough of a intra-liposome fragment to detect by immunoblot analysis. The ability for spontaneous insertion is limited by the size of the tail domain, which should not exceed 85 amino acids^18,28,45^. Our KASH5 constructs, with a tail domain of 60 amino acids, are well within this limit. As a positive control, we used a common control for spontaneous insertion assays, the cytochrome b5 (cB5) flanked with a C-terminal bovine Opsin tag^25–28^. The cB5-Opsin as well as the C-terminal V5-tagged wild-type and L535Q KASH5 were expressed in vitro using the TNT Rabbit Reticulocyte Lysate System (Promega). The in vitro expression was performed in the presence (co-translational) or absence (post-translational) of protein-free liposomes (Fig. 7A,D, respectively). For the post-translational condition, we released newly synthetized TA proteins from the ribosomes by treating lysates with puromycin prior to the addition of liposomes. After incubation with liposomes we performed a protease protection assay to determine whether the C-terminal tail had inserted into the liposomes. Half of each sample was digested with proteinase K (+ PK) and the other half was digested with proteinase K in presence of Triton X100 detergent to simultaneously permeabilize the liposomes (+ PKT) (Fig. 7A,D). As shown in the Fig. 7B,E, proteinase K treatment resulted in a 9 kDa band in both the co- and post-translational translocation assays for cB5-Opsin. This band corresponds to the predicted size of the cB5 tail with the Opsin tag. Solubilization of the liposomes with Triton X100 and proteinase K treatment resulted in the predicted loss of this band. The cB5 protein therefore inserted spontaneously into the liposomes and was protected from proteinase K degradation. However, no band (9 kDa predicted size) was detected after proteinase K treatment for the L535Q KASH5 samples, indicating that the variant KASH5 was not able to insert spontaneously in the protein-free liposomes system, either co-translationally or post-translationally (Fig. 7C,F).

## Discussion

Meiosis is an essential process for metazoans and is central to gametogenesis. The exchange of genetic information between homologous chromosomes in meiosis provides genetic variability and requires pairing and synapsis of homologues. Homologous pairing is achieved through chromosomal movements coupled with nuclear rotations, both of which occur in prophase I of meiosis and are referred to as rapid prophase movements (RPM). Any defects in homologous pairing can lead to gametogenesis failure. The SUN1/KASH5 LINC complex has been shown to be essential for RPM by connecting chromosomes to the dynein motor proteins. As shown in previous studies, loss of KASH5 in mice results in loss of nuclear rotation, chromosome movements and synapsis defects, all resulting in a spermatogenesis arrest and infertility^31,32^. Loss of SUN1 also disrupts chromosome movement and synapsis but does not completely abrogate nuclear rotation^32^. Nevertheless, SUN1-null mice are infertile^46^.

A variant of human KASH5 was recently identified in male infertility patients^40^, adding additional support to the role of KASH5 in spermatogenesis. This variant consists in a substitution of a leucine with a glutamine at the position 535 within the TMD of KASH5. The amino acid substitution changes the hydrophobicity of the TMD with a decrease of Δ6.9 kcal/mol (GES scale). In this study we show that the decreased hydrophobicity of the TMD is responsible for a mistargeting of the L535Q KASH5 away from the NE, and we suggest that this mislocalization is the mechanism by which the L535Q variant contributes to infertility. TA proteins are post-translationally targeted and generally inserted into membranes through chaperones and chaperone-associated targeting machineries^18^. The targeting of a tail-anchored protein is largely influenced by the TMD hydrophobicity as well as the tail charge. For example, a moderately hydrophobic TMD coupled with a positively charged tail promotes mitochondrial and peroxisome targeting. We show that the L535Q amino acid substitution leads to mitochondrial targeting. Interestingly, we did not observe any peroxisome localization, indicating that either the L535Q KASH5 was specifically targeted to the mitochondria membrane, or that any protein localized to the peroxisomes was efficiently cleared.

Emerging evidence indicates that the mitochondrial membrane is a default destination for TA proteins in the situation where normal targeting pathways are disrupted, or as is the case here, amino acid substitutions result in a changed hydrophobicity of the protein. To maintain its integrity and normal protein composition, mitochondria contain protein extraction mechanisms in the mitochondrial outer membrane (MOM). One of these clearing systems is ATAD1 (MSP1 in yeast), an AAA-ATPase that localizes to the mitochondria and peroxisome membranes. In association with DOA10, an ER-resident E3 ligase, ATAD1 plays a crucial role in removing and degrading mistargeted TA proteins from the MOM^47–50^. However, the mechanism by which the clearing system recognizes a mistargeted protein is not well understood^49^. The accumulation of L535Q KASH5 at the mitochondrial membrane indicates that the protein is not cleared by the quality control machinery, even at relatively low expression levels, and suggests that the protein escapes recognition by the quality control machinery. One possible explanation for this is that L535Q KASH5 may interact with a mitochondrial protein, and may therefore be seen as a *bona fide* mitochondrial protein by the clearing machinery.

It is well established that addition of amino acids to the C-terminus of KASH proteins disrupts the association with SUN proteins^7^. We were able to show that addition of an alanine to the C-terminus of L535Q KASH5 did not disrupt the mitochondrial localization. In fact, the addition of a V5 tag (14 amino acids) to the C-terminus also did not disrupt the mitochondrial localization of L535Q KASH5 (data not shown). Taken together these results suggest that if the L535Q KASH5 has a mitochondrial binding partner, the nature of this interaction is not a traditional SUN-KASH interaction. In addition, it is possible that the L535Q KASH5 does not interact with any mitochondrial proteins, but escapes clearing from the mitochondria through other unknown mechanisms.

While we are proposing that the L535Q substitution in KASH5 causes infertility by preventing RPM in meiosis, it is possible that the accumulation of L535Q KASH5 at the mitochondria could trigger mitochondrial stress and cytotoxicity leading to infertility. Indeed, our analysis of mitochondrial respiration suggests that the accumulation of L535Q at the mitochondria has a small but significant effect on mitochondrial respiration. However, this is a relatively minor effect and mitochondrial membrane potential did not seem to be affected. Loss of mitochondrial membrane potential is an early indication of mitochondrial stress^51,52^. Since we could see clear colocalization of the MitoTracker Red CMXROS signal and the GFP L535Q KASH5 signal, we suggest that mitochondrial disfunction is an unlikely contributor to the infertility caused by the variant L535Q KASH5.

In conclusion, we have investigated an infertility-associated variant in human KASH5 and shown that the amino acid substitution results in a changed TMD hydrophobicity. This change in hydrophobicity dramatically alters the localization of the KASH5 variant, away from the NE and to the mitochondria. We suggest that the absence of KASH5 at the NE is the molecular mechanism for the infertility observed in patients that are homozygous for the L535Q KASH5 variant.

## Materials and methods

### Plasmid construction and PCR-based mutagenesis

The human KASH5 cDNA was PCR amplified and subcloned into the Gateway (Invitrogen) entry vector pENTR-D-TOPO according to the manufacturer’s instruction. The KASH5 variants were generated using the Q5 Site-Directed Mutagenesis kit (NEB) following the manufacturer’s instruction. The primers used for the mutagenesis were designed with the NEBaseChanger tool (NEB) and are listed in the Table 1. All constructs were confirmed by DNA sequencing.

**Table 1:** Primers list for mutagenesis.

L535Q	*Fwd: CTGCTGCTGCAGCTCTCTGTCC*
	*Rev: CAGCAGGCCCAGGACAGG*
L535V	*Fwd: GCTGCTGCTGGTGCTCTCTGTC*
	*Rev: AGCAGGCCCAGGACAGGA*
L535N	*Fwd: GCTGCTGCTGAATCTCTCTGTCCTGCTG*
	*Rev: AGCAGGCCCAGGACAGGA*
L530Q	*Fwd: GTCCTGGGCCAGCTGCTGCTG*
	*Rev: AGGAGCTGGGATCAGTGGATG*
L531Q	*Fwd: CTGGGCCTGCAGCTGCTGCTG*
	*Rev: GACAGGAGCTGGGATCAGTGG*
L532Q	*Fwd: GGGCCTGCTGCAGCTGCTGCTG*
	*Rev: AGGACAGGAGCTGGGATCAGT*
L533Q	*Fwd: CTGCTGCTGCAGCTGCTGCTCT*
	*Rev: GCCCAGGACAGGAGCTGGG*
L534Q	*Fwd: CTGCTGCTGCAGCTGCTCTCTG*
	*Rev: CAGGCCCAGGACAGGAGC*
L536Q	*Fwd: CTGCTGCTGCAATCTGTCCTGCTGCTT*
	*Rev: CAGCAGCAGGCCCAGGAC*
L535Q + Ala	*Fwd: GCATGAAAGGGTGGGCGCGCCG*
	*Rev: CACTGGAGGGGGCTGGAG*
WT + Ala	*Fwd: GCATGAAAGGGTGGGCGCGCCG*
	*Rev: CACTGGAGGGGGCTGGAG*

To produce the expression plasmids, the various pENTER clones were transferred into destination vectors using the LR recombination reaction (Invitrogen). The following destination vectors were used: pcDNA6.2/N-GFP-DEST (Invitrogen) for N-terminal GFP-tagged proteins; pcDNA3.2/V5-DEST (Invitrogen) for C-terminal V5-tagged proteins. All constructs were verified by DNA sequencing after recombination. DNA for transfection was prepared by EndoFree Plasmid Maxiprep (Qiagen). The cytochrome B5 (cB5) plasmid was a kind gift from Professor Stephen High (University of Manchester)^53,54^.

### Cell lines and transfection

U2OS and MCF-7 cells (#HTB-96 and HTB-96, respectively from ATCC) were cultured in a humidified incubator at 37 °C and 5% CO_2_ in Dulbecco’s modified Eagle’s medium supplemented with 10% fetal bovine serum (FBS), 100 μg/ml streptomycin, 100 U/ml penicillin and 2 mM l-glutamine. All cell transfections were performed in 6-well plates with a total of 0.3 μg DNA/well using Effectene transfection reagent (Qiagen) following the manufacturer’s instructions.

### Immunofluorescence, confocal and colocalization studies

Cells were seeded on coverslips in 6-well plate at 70% confluency and were transfected 24 h after seeding. After 48 h, cells were fixed in 4% paraformaldehyde (PFA) in phosphate-buffered saline (PBS) for 10 min at room temperature (RT), permeabilized with 0.2% Triton X-100 in PBS for 5 min and blocked in blocking buffer (10% donkey normal serum, 0.2% Triton X-100 in PBS) for 1 h at RT. Samples were incubated in primary antibody solution (diluted in blocking buffer) for 1 h at RT. The primary antibodies used were as follows: mouse anti-TOM20 (1/200, #sc-17764, Santa Cruz Biotechnology Inc.), rabbit anti-Pex14 (1/200, #ab183885, Abcam) and mouse anti-Lamin A/C (1/250, #MA3-1000, ThermoScientifc). Coverslips were incubated for one hour in secondary antibody diluted in blocking buffer supplemented with 1 µg/ml 4,6-diamidino-2-phenylindole (Dapi, ThermoScientific). Secondary antibodies were as follows: donkey anti-rabbit Alexa Fluor 568 (1/500, #A10042, Invitrogen), donkey anti-mouse Alexa Fluor 568 (1/500, #A10037, Invitrogen) and donkey anti-mouse Alexa Fluor 647 (1/500, #A31571, Invitrogen). Coverslips were mounted on slides using ProLong Gold anti-fade mounting medium (Invitrogen). All steps above were interspersed by PBS washes.

To label mitochondria with the MitoTracker Red CMXRos, cells were incubated with 50 nM MitoTracker Red CMXRos (M7512, Thermo Fisher Scientific) in FBS-free DMEM for 20 min at 37 °C with 5% CO_2_. Cells were then washed with warm PBS and fixed with 4% PFA in PBS for 10 min at RT.

Images were acquired on a Nikon A1 confocal microscope with a 60 × oil immersion objective or with a Zeiss confocal microscope with a 63 × oil immersion objective. Image analysis was performed with ImageJ.

Colocalization of GFP-KASH5 with organelle markers was calculated with the Pearson’s correlation coefficient using the NIS software. The number of cells used for each experiment is indicated in the figure legends. For each cell, a region of interest (ROI) encompassing the organelle marker signal was drawn manually. Analysis was restricted to the Lamin A/C signal for the nucleus, TOM20 for the mitochondria and PEX14 for the peroxisome. The Pearson’s correlation coefficient was calculated between the green (GFP-KASH5) and the red or deep red channels (organelle marker). For statistical analysis, the experiments were repeated at least four times.

### Oxygen consumption rate

Measurement of oxygen consumption rate were performed using a Seahorse Bioscience XF96 instrument (Seahorse Biosciences) according to the manufacturer’s instructions. Briefly, U2OS cells were seeded into the Seahorse 96-well plate at a density of 25,000 cell/well, followed by culturing for 24 h. The cells were equilibrated in a non-CO_2_ incubator for 60 min prior to the assay. After that, oligomycin (1 μM), carbonyl cyanide p-trifluoromethoxyphenylhydrazone (FCCP, 2 μM) and rotenone/antimycin A (1 μM) were added into the A, B and C ports of the Seahorse cartridge, respectively. Data were expressed as the oxygen consumption rates (OCR; pmol/min). Readings were normalized against cell numbers as follows: after the Seahorse reading was completed, cells were incubated with 1 µg/mL Hoechst 33,342 for 30 min at 37 °C with 5% CO_2_ and read on a FlexStation 3 Microplate Reader (Molecular Devices). The average fluorescence value for the control wells was used to obtain the normalization factor for all wells. The results are the average of two biological replicates and 30 technical replicates for each sample.

### Determination of the membrane potential

Cells were seeded in a 6 cm plate at 70% confluency and were transfected with GFP-L535Q KASH5 24 h after seeding. After 24 h, cells were plated into 16 wells of a 48-well plate. After another 24 h, control cells were treated with 20 μM carbonyl cyanide p-trifluoromethoxyphenylhydrazone (FCCP) in DMEM for 10 min at 37 °C with 5% CO_2_. After the treatment, 200 nM tetramethylrhodamine ethyl ester (TMRE) was added to the cells for 30 min at 37 °C with 5% CO_2_. Cells were then washed with warm 0.2% BSA/PBS and imaged immediately. Images were acquired on an EVOS (ThermoFisher Scientific) cell imaging system. This experiment was repeated three times.

### In vitro translation/transcription (IVT) assay and translocation assay

In vitro proteins were synthetized using the T7 TNT Quick Coupled Transcription/Translation System (Promega) according to the manufacturer’s protocol. For co-translational reaction, IVT reaction was performed in presence of protein-free liposomes for 90 min at 30 °C. For post-translational condition, the IVT reaction was terminated after 90 min with the addition of 1 mM puromycin for 20 min at 30 °C to release the neo-synthetized proteins from the ribosomes. Thereafter, the liposomes were added to the post-translational IVT reaction and the samples were incubated for 2 h at 32 °C.

### Protease protection assay

The protease protection assay for in vitro synthetized proteins was performed after the translocation assay. Samples were divided by volume into three equal aliquots: the first was untreated, the second was treated with 0.25 mg/ml proteinase K (+ PK) and the third was treated with 0.25 mg/ml proteinase K in presence of 0.2% TritonX100 (+ PKT). After 30 min incubation on ice, the proteinase K activity was inhibited with 10 mM phenylmethylsulfonyl fluoride (PMSF) for 5 min on ice. Samples were then diluted into 10 volumes of 1% SDS, 0.1 M Tris (pH 8) and incubated at 95 °C for 10 min.

### Protein-free liposomes preparation

Liposomes were prepared from a 20 mg/ml mixture of phospholipids (Avani Polar Lipids) containing: phosphatidyl choline 54%; phosphatidyl ethanolamine 27%; phosphatidyl inositol 13%; phosphatidyl serine 2% and cardiolipin 0.3%.

The method of liposome preparation was as previously described^27^. Briefly, lipids were dissolved with a 2:1 mixture of chloroform:methanol. The organic solvent was evaporated under a stream of nitrogen and dessicated overnight under vacuum. Thereafter, the lipid film was resuspended at 10 mg/ml by overnight mixing with a solution containing 15% Glycerol, 50 mM HEPES pH 7.5 and 10 mM Dithiothreitol (DTT). To homogenize liposome size, we extruded the lipid suspensions at 50 °C ten times through a 100 nm polycarbonate nucleopore membrane using an Avanti mini-extruder. Liposomes were then sedimented at 70,000 RPM for 30 min. The liposomes pellet was resuspended in 20 µl of storage buffer (50 mM HEPES pH 7.5, 100 mM KAc, 250 mM sucrose, 2 mM MgCl2 and 1 mM DTT) and aliquoted as single use aliquots. Aliquots were flash-frozen in liquid nitrogen and stored at − 80 °C.

### Western blot analysis

Proteins were resolved by SDS-PAGE and transferred onto PVDF membranes. Membranes were blocked with 5% milk in TBS with 0.1% Tween20 (TBST) for 1 h at RT. Primary antibody incubations were performed overnight at 4 °C with primary antibodies diluted in 5% milk-TBST. The primary antibodies used were as follows: mouse anti-V5 (1/1,000, ThermoScientific), mouse anti-Opsin (1/1,000, a kind gift from Professor Stephen High^55^). HRP anti-rabbit or HRP anti-mouse secondary antibodies (1/10,000, #115-035-003 Jackson) were used for detection. Finally, the membranes were incubated with ECL detection (SuperSignal West Dura, ThermoFisher Scientific) and visualized with a ChemiDoc system (BioRad).

### Statistical analysis

Colocalization was analyzed using the Pearson’s correlation coefficient. Significance was calculated using Mann–Whitney tests. P values less than 0.05 were considered to indicate statistical significance. Pearson’s correlation values are presented as mean ± SD.

## Supplementary Information

Supplementary Information.
