# Supplementary material for: A human infertility-associated KASH5 variant promotes mitochondrial localization
Source: Sci Rep. 2021 May 12;11:10133. doi: 10.1038/s41598-021-89439-2 (PMC8115505; doi:10.1038/s41598-021-89439-2)
Supplement: Supplementary file 1 — Supplementary Information. [file 41598_2021_89439_MOESM1_ESM.docx]

Supplementary information

**A human infertility-associated KASH5 variant promotes mitochondrial localization.**

Sana A. Bentebbal^1^, Bakhita R. Meqbel^1^, Anna Salter^2,3^, Victoria Allan^2^, Brian Burke^3^, Henning F. Horn^1*^.

^1^College of Health and Life Sciences, Hamad Bin Khalifa University, Doha, Qatar.

^2^Faculty of Life Sciences, University of Manchester, Manchester, M13 9PT, UK.

^3^Laboratory of Nuclear Dynamics and Architecture, Institute of Medical Biology, Agency for Science, Technology and Research (A*STAR), Singapore.

**Figure S1. L535Q KASH5 localizes at the mitochondria**

HeLa (**A**) and MCF-7 (**B**) cells transfected with a GFP-tagged version of wild-type or L535Q KASH5 and treated with Mitotracker Red CMXROS to stain the mitochondria. (**C**) MCR-5 cells co-transfected with a GFP-tagged version of wild-type or L535Q KASH5 and a mito-dsRed construct that localizes to the mitochondria. The wild-type KASH5 localizes around the nucleus whereas the L535Q KASH5 localizes at the mitochondria.

(**D**) Isolated mitochondria from U2OS cells transfected with a GFP-tagged version of wild-type or L535Q KASH5. The mitochondria fraction was analyzed by immunoblot assay using an anti-GFP antibody to detect KASH5 protein. Anti-COXIV and anti-H3 antibodies were used as markers for the nuclear and mitochondrial extracts.

**Figure S2. L535Q KASH5 does not alter mitochondria membrane potential.** Membrane potential was assessed with the TMRE dye staining. U2OS cells transfected with a GFP-tagged version of L535Q KASH5 were treated or not with 10μM FCCP, an uncoupler of mitochondrial oxidative phosphorylation, prior to TMRE staining.
